# Climate Change and West Nile Virus in a Highly Endemic Region of North America

**DOI:** 10.3390/ijerph10073052

**Published:** 2013-07-22

**Authors:** Chen C. Chen, Emily Jenkins, Tasha Epp, Cheryl Waldner, Philip S. Curry, Catherine Soos

**Affiliations:** 1 Large Animal Clinical Sciences, Western College of Veterinary Medicine, University of Saskatchewan, 52 Campus Drive, Saskatoon, Saskatchewan S7N 5B4, Canada; E-Mails: tasha.epp@usask.ca (T.E.); cheryl.waldner@usask.ca (C.W.); 2 Department of Veterinary Microbiology, Western College of Veterinary Medicine, University of Saskatchewan, 52 Campus Drive, Saskatoon, Saskatchewan S7N 5B4, Canada; E-Mail: emily.jenkins@usask.ca; 3 Saskatchewan Ministry of Health, 3475 Albert Street, Regina, Saskatchewan S4S 6X6, Canada; E-Mail: pcurry@health.gov.sk.ca; 4 Environment Canada, Science & Technology Branch, 115 Perimeter Road, Saskatoon, Saskatchewan S7N 0X4, Canada; E-Mail: catherine.soos@ec.gc.ca

**Keywords:** West Nile virus, *Culex tarsalis*, climate change, Canadian prairies, spatial and temporal distribution, habitat

## Abstract

The Canadian prairie provinces of Manitoba, Saskatchewan, and Alberta have reported the highest human incidence of clinical cases of West Nile virus (WNV) infection in Canada. The primary vector for WVN in this region is the mosquito *Culex tarsalis.* This study used constructed models and biological thresholds to predict the spatial and temporal distribution of *Cx. tarsalis* and WNV infection rate in the prairie provinces under a range of potential future climate and habitat conditions. We selected one median and two extreme outcome scenarios to represent future climate conditions in the 2020 (2010–2039), 2050 (2040–2069) and 2080 (2070–2099) time slices. In currently endemic regions, the projected WNV infection rate under the median outcome scenario in 2050 raised 17.91 times (ranged from 1.29-27.45 times for all scenarios and time slices) comparing to current climate conditions. Seasonal availability of *Cx. tarsalis* infected with WNV extended from June to August to include May and September. Moreover, our models predicted northward range expansion for *Cx. tarsalis* (1.06–2.56 times the current geographic area) and WNV (1.08–2.34 times the current geographic area). These findings predict future public and animal health risk of WNV in the Canadian prairie provinces.

## 1. Introduction

Climate conditions, such as temperature and precipitation, are among many important factors that determine the spatial and temporal distribution of vectors and vector-borne diseases. Changes in climate influence the occurrence of vector-borne diseases in the following three major ways: (a) reproduction, development, and survival of vectors, which in turn drive the distribution and abundance of vectors; (b) blood seeking activity of vectors; and c) rates of pathogen amplification, through development, multiplication, and survival within vectors [1,2]. In addition, climate conditions may affect the distribution, abundance, behavior, phenology of reproduction, and migration of vertebrate hosts [3,4]. Therefore, climate change will drive dramatic alterations in the spatial and temporal distribution and overall incidence of vector-borne diseases. Besides these direct effects of climate change on vector-borne diseases, climate change can also lead to substantial alterations in landscape, which in turn influence the distribution and abundance of hosts, vectors, and vector-borne pathogens [1,5]. Without taking these ecological effects of climate change and their interactions into consideration, projections of the potential effects of climate change on vector-borne diseases will remain inaccurate [1,5]. 

**Figure 1:**
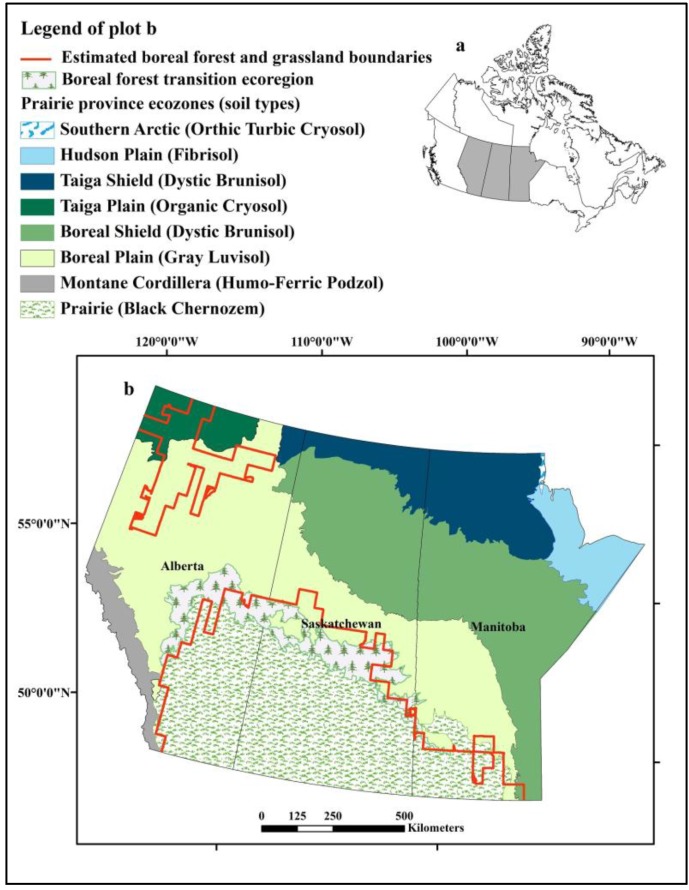
Distribution of ecozones and soil types in the prairie provinces of Alberta, Saskatchewan, and Manitoba, Canada and the boreal forest and prairie boundaries estimated using baseline climate conditions. (**a**) Location of prairie provinces (grey shading) in Canada. (**b**) Enlargement of Prairie provinces and distribution of ecozones.

West Nile virus (WNV) from the family Flaviviridae, genus Flavivirus was introduced into the Western Hemisphere in 1999 [6]. Since that time the Canadian prairies, grassland ecozone in the southern parts of the provinces of Manitoba, Saskatchewan, and Alberta (Figure 1), have generally had the highest human incidence of clinical cases of WNV infection in Canada. During the 2007 epidemic season, a total of 2,215 confirmed clinical cases of WNV infection were reported in Canadians, of which 98% occurred in the prairie provinces, including 1,285 in Saskatchewan, 578 in Manitoba, and 318 in Alberta [7]. As a newly introduced vector-borne disease affecting a wide range of vertebrate hosts, WNV remains a significant concern for public health and wildlife conservation in the Canadian prairies.

In the Canadian prairies, the mosquito species *Culex tarsalis* Coquillett is the principal vector for WNV [8,9] This mosquito species is one of the most competent WNV vectors evaluated to date in laboratory studies [10] and is the predominant potential vector species in the Canadian prairies during the summer WNV season [8]. The southern boundary of the boreal forest transition zone is identified as the northernmost limit of WNV distribution in western North America [8], although *Cx. tarsalis* has been recorded further north [11]. Climate, particularly temperature and precipitation, and habitat preference determine the distribution of *Cx. tarsalis* in western North America [12]. Grassland and agriculture area are the preferred land cover type for *Cx. tarsalis* in the Canadian prairies [8,9,13] and other regions of the Great Plains [12,14]. Stagnant water bodies with high organic content are favored sites for oviposition by *Cx. tarsalis* [8,15]. In the Canadian prairies, larvae of *Cx. tarsalis* are commonly found in many temporary water bodies, such as artificial containers, water-filled hoof prints, and weedy roadside ditches [8,12]. Furthermore, large water bodies and running water are not suitable for larval development due to the disturbance and lower nutrition concentration [16]. Studies have found that the percentage of wetland is not associated with the abundance of *Cx. tarsalis* and WNV risk in the Canadian prairies and northern Great Plains [13,14,17,18]. 

Several biological features of *Cx. tarsalis* facilitate the transmission of WNV including its capacity to: vertically transmit WNV to its offspring [19]; produce multiple generations per season; and take multiple blood meals during each generation [8]. Because it feeds on both avian and mammalian hosts, *Cx. tarsalis* plays the role of a “bridging vector” transmitting WNV from its enzootic cycle to humans and other mammalian species [20,21,22]. Besides WNV, *Cx. tarsalis* is the primary competent vector for the St. Louis and Western Equine Encephalitis viruses in North America [8,23,24].

Changes in future climate will not only influence the distribution of vectors and pathogens, but also the habitat suitability for vectors [25]. Therefore, the assessment of possible effects of climate change on grassland distribution is critical for predicting the occurrence of *Cx. tarsalis* and WNV under future climate change. In a warmer and possibly drier future climate, current grassland habitat in the Canadian prairie ecozone might be replaced by the grassland flora found in the United States, and the boreal forest in the northern prairie provinces might be replaced by aspen parkland and grassland [26,27,28]. The southern boundary of the boreal forest fits very closely with the zero isoline of the annual climate moisture index, estimated by mean annual precipitation minus potential evapotranspiration (PET) [29]. 

In the present study, we integrated empirically derived, biologically-relevant temperature thresholds for *Cx. tarsalis* survival and WNV development, and statistical models in order to predict the effects of climate change on the distribution and abundance of *Cx. tarsalis* and WNV in the Canadian prairies, one of the most highly endemic regions in North America. Furthermore, we took into account potential changes in landscape as a result of climate change, including predicting the distribution of grassland habitat under future climate changes [26,27]. Our objectives were to assess and predict the potential effects of climate change on the abundance of *Cx. tarsalis* and infection rate of WNV in *Cx. tarsalis* in the Canadian prairie ecozone, under the assumption that competent avian amplifying hosts will continue to persist in this region. In addition, we explored the possibility of northward expansion of *Cx. tarsalis* and WNV out of their current distribution area in the Canadian prairie ecozone.

## 2. Materials and Methods

### 2.1. Selection of Three Climate Change Outcome Scenarios

Monthly climatology data between 1961and 1990 were downloaded from the CRU Global climate data set (IPCC Data Distribution Centre; http://www.ipcc-data.org/; accessed in December, 2012) to represent the 30-year baseline climate conditions of the prairie provinces [30]. For creating outcome scenarios representing a wide array of future climate conditions in the prairie provinces, we considered a total of 142 experiments in three future emission scenarios (SRA2, SRA1B, SRB1) constructed using 24 general circulation models (GCMs) [31] (Table 1). These were selected on criteria of plausibility and best international standards of practice at the time. Experiments of each emission scenario represent results of simulations that assume a forcing of 1% per year in equivalent CO_2_ concentration, radiative forcing and variably include aerosol effects. Future emission scenarios of greenhouse gases and aerosols in the atmosphere depend on factors such as population and economic growth and energy use [30].

Average changes with respect to 1961–1990 were calculated for the 30 year periods centered on the 2020s (2010–2039), 2050s (2040–2069) and 2080s (2070–2099) for each of the 142 experiments. In order to select three outcome scenarios, representing cool and wet, median, and warm and dry climate conditions in the prairie provinces, scatter plots of changes in mean temperature and precipitation associated with 142 experiments were created for the study area. The 2050s time slice was used to select the three outcome scenarios; by the 2080s, the magnitude of uncertainty in results increased substantially. The selection of representative outcome scenarios (median and range) followed the guidelines put forward by the Intergovernmental Panel on Climate Change Task Group on Data and Scenario Support for Impact and Climate Assessment [30]. 

**Table 1:** Experiments considered in the current study under given General Circulation Models and emissions scenarios to select future median and extreme climate scenarios for the Canadian Prairies.

General circulation models	SRA2	SRA1B	SRB1	Resolution Latitude (°)	Resolution Longitude (°)
BCCR-BCM2.0	1	1	1	1.9	1.9
CGCM3.1_T47	5	5	5	2.8	2.8
CGCM3.1_T63	1	1	1	1.9	1.9
CNRM-CM3	1	1	1	1.9	1.9
CSIROMk3.0	1	1	1	1.9	1.9
CSIROMk3.5	1	1	1	1.9	1.9
ECHAM5	3	4	3	1.9	1.9
ECHO-G	3	3	3	3.9	3.9
FGOALS		3	3	2.8	2.8
GFDL-CM2.0	1	1	1	2.0	2.5
GFDL-CM2.1	1	1	1	2.0	2.5
GISS-AOM		2	2	3.0	4.0
GISS-EH		3		4.0	5.0
GISS-ER (run number) ^1^	1 (1)	2 (2, 4)	1 (1)	4.0	5.0
INGV-SXG	1	1		1.1	1.1
INM-CM3.0	1	1	1	4.0	5.0
IPSL-CM4	1	1	1	2.5	3.75
MIROC3.2-hires		1	1	1.1	1.1
MIROC3.2-medres	3	3	**3** **(run 2)**	2.8	2.8
CGCM2.3.2	5	5	5	2.8	2.8
NCAR-CCSM (run numbers)	4 (1–4)	7 (1–3, 5–7, 9)	8 (1–7, 9)	1.4	1.4
NCAR-PCM	4	4	**3** **(run 2)**	2.8	2.8
UKMO-HadCM3	1	1	1	2.5	3.75
UKMO-HadGEM1	1	**1**		1.3	1.9
Total experiments	40	54	48		

^1^ Run number of the experiments used in the current study. Bold text indicates the experiments selected as median and extreme climate outcome scenarios in the current study.

Future climate for each variable, such as monthly mean temperature and monthly total precipitation, was computed by combining baseline climate condition and predicted changes for each of the three outcome scenarios and the three time slices.

### 2.2. Models for Cx. tarsalis Abundance and WNV Infection Rate

Recorded data on abundance of *Cx. tarsalis* and WNV infection rate were obtained from mosquito trapping in the prairie provinces from May to September for 2005 to 2008 from the Public Health Agency of Canada, Alberta Environment, Manitoba Public Health and Healthy Living, and Saskatchewan Ministry of Health. Counts of *Cx. tarsalis* per trap site per night were transformed by ln(y+1) to normalize the data distribution prior to analysis [32]. *Culex tarsalis* infection rate (defined as the number of mosquitoes infected with WNV in 1000 pooled mosquitoes) was computed using PooledInfRate (version 3.0), a Microsoft® Excel plug-in [33] by Maximum Likelihood (ML-IR) and minimum infection rate (MIR) methods [33,34]. 

Two models using temperature and precipitation as the primary explanatory variables were constructed to predict the abundance of *Cx. tarsalis* and WNV infection rate in *Cx. tarsalis* in the Canadian prairies (Statistical Analysis System, version 9.2, Cary, NC, USA), details of which are published in Chen *et al.* (in review). Briefly, a linear mixed model (PROC MIXED, SAS Institute 2008) was used to predict abundance of* Cx. tarsalis*. Parameters were estimated by the restricted maximum likelihood method. We then adopted a generalized linear mixed model, with a log link function (PROC GLIMMIX, SAS Institute 2008) for prediction of *Cx. tarsalis* infection rate. A negative binomial distribution was chosen in the *Cx. tarsalis* infection rate model based on a preliminary analysis where the formula ‘Pearson Chi-Square divided by degrees of freedom (DF)’ value was close to one and the lowest Akaike information criterion (AIC) value. A summary of the final *Cx. tarsalis* abundance model and the WNV infection rate model, as well as parameter coefficients, are shown in Table 2.

**Table 2:** Coefficients of variables in the final models of *Cx. tarsalis* abundance and WNV infection rate.

Variables (unit)	*Cx. tarsalis* abundance		WNV infection rate	
	Coefficient	95% CI	Coefficient	95% CI
Intercept	−3.48	−4.05 to −2.91	−2.26	−4.47 to −0.05
*Cx. tarsalis* abundance (log(y+1))			0.55	0.31 to 0.79
**Climate variables**				
Monthly mean temperature (1 °C)	0.22	0.2 to 0.25		
1 month lagged temperature (1 °C)	0.07	0.05 to 0.09	0.32	0.22 to 0.41
3 months total of monthly mean degree days (dd)			−0.10	−0.2 to −0.01
Monthly total precipitation (1 mm)	0.0033	0.002 to 0.005		
1 month lagged precipitation (1 mm)	0.0042	0.003 to 0.005	−0.27	−0.36 to −0.18
2 month lagged precipitation (1 mm)	0.0033	0.002 to 0.004		
3 months total precipitation (1 mm)			−0.05	−0.08 to −0.02

CI: confidence interval.

### 2.3. Modeling Grassland Distribution

We used models constructed by Hogg [35] and simplified Penman-Monteith method to predict the future boundaries of the boreal forest and grassland in the prairie provinces [35]. The predicted distribution of grassland habitat was used as a criterion for the occurrence of *Cx. tarsalis* under the three selected future outcome scenarios. To estimate the PET, a series of conditions and corresponding equations simplified from the Penman-Monteith method [35] were derived as follows:

For *T*mean > 10 °C: PET = 93 *D* exp(A/9300)
For 10 °C ≥ *T*mean > –5 °C: PET = (6.2*T*mean + 31) *D* exp(A/9300)
For *T*mean ≤ –5 °C: PET = 0
*T*mean = mean monthly temperature (unit: °C)
*D* = vapour pressure deficit (unit: KPa)
A = altitude (unit: meter)

The vapour pressure deficit (*D*) was calculated using this formula:

*D* = 0.5 (*e*_Tmax _+ *e*_Tmin_) – *e*_Tdew_
*e*_Tmax _= saturated vapor pressure at the maximum monthly mean temperature
*e*_Tmin_ = saturated vapor pressure at the minimum monthly mean temperature
*e*_Tdew_ = saturated vapor pressure at the dew point temperature.

The *e*_Tdew_ was set as equal to the saturation vapour pressure at a temperature of 2.5 °C lower than mean minimum temperature [35].

### 2.4. Maps of Current and Future Distribution

Using these models and the baseline and projected climate conditions in the prairie provinces, we used ArcGIS version 10 (Environmental System Research Institute, Redlands, CA, USA) to create maps of current and future abundance of* Cx. tarsalis* and WNV infection rate.

#### 2.4.1. Maps of *Cx. tarsalis* Abundance

According to the thermal tolerance limits and habitat requirements for *Cx. tarsalis*, the occurrence of *Cx. tarsalis* was set as zero when the monthly mean temperature was lower than 14 °C or higher than 35 °C [36], or the habitat was not grassland. The constructed *Cx. tarsalis* abundance model (Table 2) was then populated with current and future climate conditions under the three outcome scenarios to map the monthly abundance and distribution of *Cx. tarsalis* in the Canadian prairie provinces.

#### 2.4.2. Maps of WNV Infection Rate in *Cx. tarsalis*

In the WNV infection rate model, WNV transmission was considered possible when the following criteria were met: (a) the primary vector, *Cx. tarsalis*, was present; (b) the temperature was higher than 14.3 °C, the minimum temperature for WNV amplification to occur in *Cx. tarsalis* [37]; and (c) at least 82 degree days were accumulated in a 12 day feeding period of the life cycle for a female *Cx. tarsalis.* This represents the minimum amount of warming needed to complete the extrinsic incubation period of the virus in the mosquito [38,39]. The WNV infection rate model was then populated with current and future climate conditions under the three outcome scenarios to predict distribution and rates of WNV infection in *Cx. tarsalis* in the study region.

## 3. Results

### 3.1. Selection of Three Climate Change Outcome Scenarios

Based on predicted changes in mean temperature and precipitation in the 2050s, three outcome scenarios representing three GCMs and two emissions scenarios were selected to represent future of cool and wet, median, and warm and dry climate conditions in the Canadian prairie provinces (Table 3). Average temperature was predicted to increase by 1–7 °C for all months in all selected outcome scenarios and time slices (Table 3). Although average annual total precipitation was projected to increase (by 21–46 mm) in all selected outcome scenarios and time slices, changes in precipitation varied among different months and areas in the prairie provinces. Under the warm and dry outcome scenario, the largest decreases in monthly precipitation occurred in July and August, with decreases by over 50% in some areas.

**Table 3:** Outcome scenarios selected to represent the range of effects of future climate change in the Canadian prairie provinces and their associated changes in mean annual temperature and precipitation in 3 time slices, compared to baseline climate conditions (1961–1990).

Experiments and time slices	Emissions Scenarios	Outcome Scenarios	Change in annual total precipitation (SD); mm	Change in mean annual temperature (SD); °C
**2010–2039 (2020s)**				
NCAR-PCM run 2	B1	Cool, wet	22.4 (8.9)	1.14 (0.27)
MIMR	B1	Median	25.8 (16.3)	1.63 (0.18)
UKMO-HadGEM1 run 1	A2	Warm, dry	44.2 (16.9)	1.65 (0.40)
**2040–2069 (2050s)**				
NCAR-PCM run 2	B1	Cool, wet	41.1 (12.8)	1.77 (0.28)
MIMR	B1	Median	37.9 (28.1)	3.04 (0.11)
UKMO-HadGEM1 run 1	A2	Warm, dry	21.3 (22.9)	4.03 (0.39)
**2070–2099 (2080s)**				
NCAR-PCM run 2	B1	Cool, wet	52.3 (12.6)	2.42 (0.33)
MIMR	B1	Median	46.4 (31.2)	4.24 (0.19)
UKMO-HadGEM1 run 1	A2	Warm, dry	29.4 (31.4)	6.80 (0.50)

SD: standard deviation.

### 3.2. Grassland Distribution

**Figure 2:**
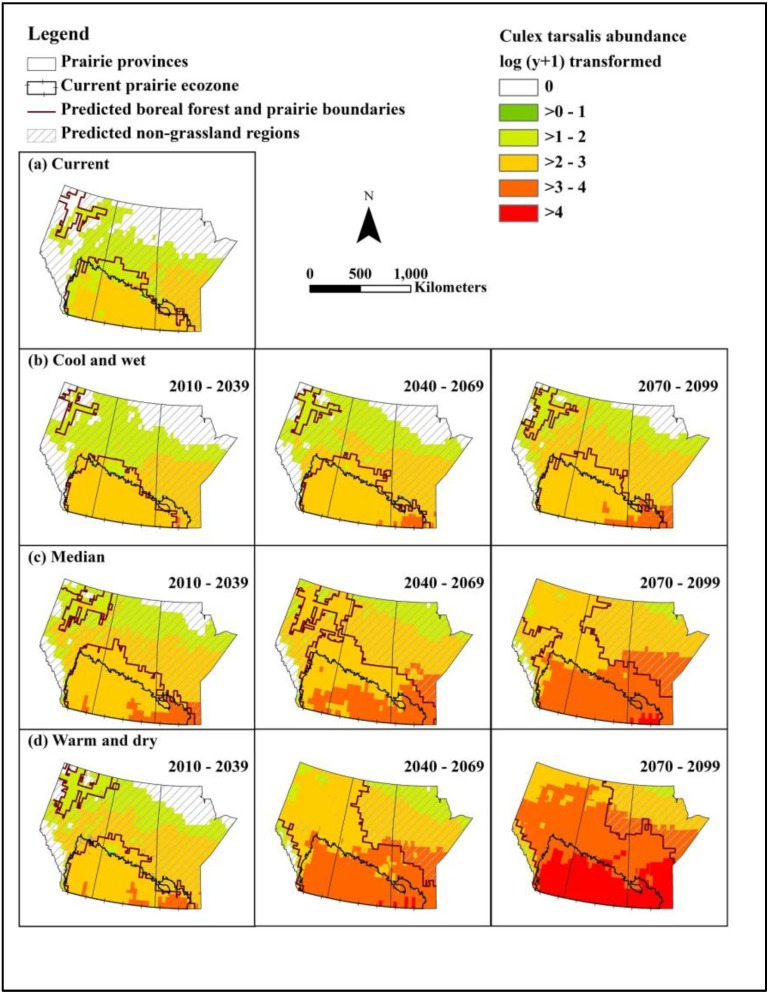
Projected spatial distribution and abundance, log (y+1) transformed, of *Cx. tarsalis* in August in the prairie provinces under current and selected outcome scenarios in three future time slices. The possible spatial distribution of *Cx. tarsalis* restricted by the predicted grassland distribution is indicated by the solid red line.

Prediction of grassland distribution showed two main areas where climate would be appropriate for grassland under current and proximal future climate scenarios. Under current conditions, the principal area of grassland habitat was the Canadian prairie ecozone, and a second, smaller patch was located in northern Alberta (Figure 1). Under the cool and wet future outcome scenario, the overall area of grassland habitat decreased by 23,004 km^2^and 7,762 km^2^ in the 2020s and 2050s, respectively, with an expansion of 57,132 km^2 ^in the 2080s (Table 4, Figure 2). In the median and warm, dry scenarios, grassland habitat expanded in all time slices (298,683 km^2 ^expansion for the median scenario in 2050s, range 17,765–842,110 km^2^), and furthermore, the two regions of grassland habitats merged into a large grassland by the 2050s (Table 4, Figure 2).

### 3.3. Culex tarsalis Abundance and Distribution

Increases in the following factors were associated with an increase in abundance of *Cx. tarsalis* in the model: mean temperature, one month lagged mean temperature, monthly total precipitation, and one and two months lagged total precipitation. The predicted warmest mean temperature for all of the selected outcome scenarios and time slices was lower than the upper threshold (35 °C) for survival of adult female *Cx. tarsalis*. The temporal distribution of *Cx. tarsalis* under current climate conditions was restricted to a period from June to August, with highest abundance in July and August (Table 5). Under future climate conditions, the temporal occurrence of *Cx. tarsalis* was extended between May and September for all selected outcome scenarios by the 2080s, and for all but the cool wet scenario by the 2050s. Furthermore, abundance of *Cx. tarsalis* was predicted to increase 1.4 times under the median outcome scenario in the 2050s (1.1 times under the cool, wet scenario in the 2020s, and 2 times under the warm, dry scenario in the 2080s) in the Canadian prairie ecozone compared to baseline climate conditions (Table 5, Figure 2).

Projected spatial distribution showed that the highest abundance of *Cx. tarsalis* occurred in the southern part of the Canadian prairies under baseline climate conditions and all selected future outcome scenarios (Figure 2). Except for the cool and wet scenario in the 2020s, the distribution of *Cx. tarsalis* expanded northward under the future outcome scenarios and time slices. The expansion of geographical distribution of *Cx. tarsalis* was 33,195 km^2^ (1.60 fold increase) under the median scenario in the 2050s (no change under the cool, wet scenario in the 2020s and a 3 fold increase under the warm, dry outcome scenario in the 2080s) (Table 4, Figure 2). Climate conditions in the northern parts of the prairie provinces (up to 60° N latitude, currently parkland and boreal forest habitats) were suitable for *Cx tarsalis* under current and future climate scenarios and therefore, the northward expansion of *Cx. tarsalis* will be primarily restricted by the absence of suitable grassland habitat (Figure 2).

### 3.4. WNV Distribution and Infection Rate in Cx. tarsalis

Under baseline climate conditions, the current temporal distribution and transmission season of WNV was limited to a period between June and August in the Canadian prairies. August was the month with the highest mean WNV infection rate of* Cx. tarsalis* (Table 6). The temporal occurrence of WNV in the Canadian prairie ecozone was extended from the current months of June to August to include May and September in all selected future outcome scenarios by the 2080s, and all but the cool and wet outcome scenario in the 2050s (Table 6). Compared to baseline, the August infection rate for the median scenario in the 2050s increased 18 fold (1.3 fold change under the cool, wet scenario in the 2020s and 27 fold change under the warm, dry scenario in the 2080s) (Table 6). 

**Figure 3:**
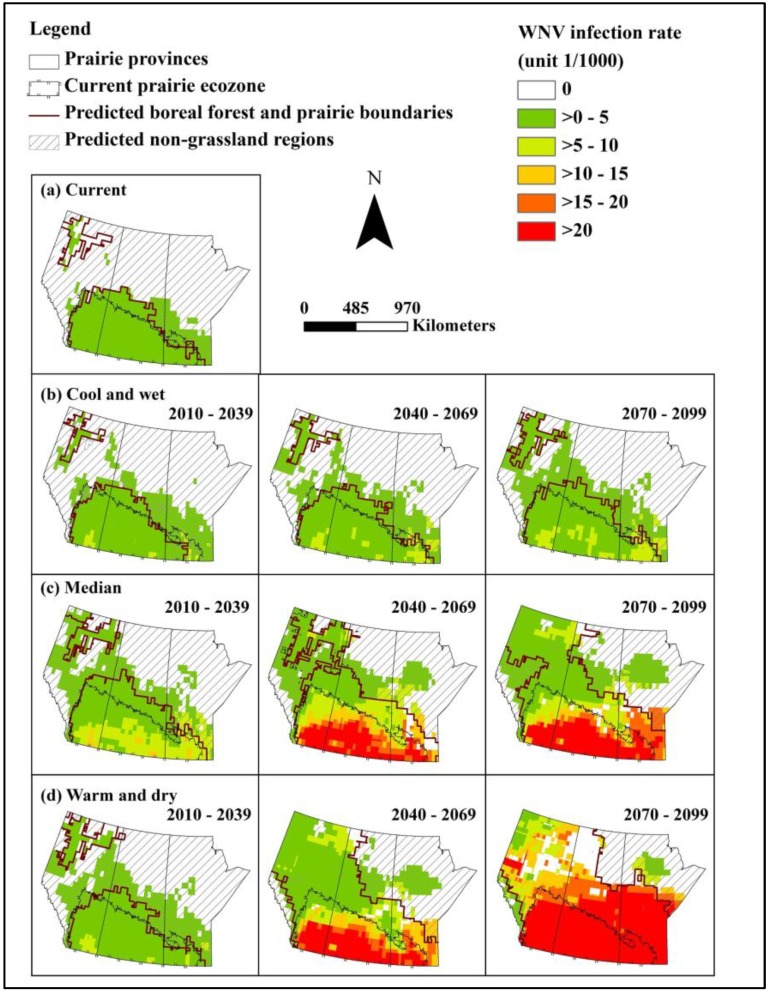
Projected WNV infection rate in *Cx. tarsalis* in August in the prairie provinces under the current (1961–1990) and selected outcome scenarios in three future time periods. The possible spatial distribution of WNV restricted by the predicted grassland distribution is indicated by the solid red line.

The projected future WNV distribution showed a decrease in distribution area of 23,258 km^2^ in the cool and wet outcome scenario in the 2020s, due to decreased area of grassland habitat. However, in all other future outcome scenarios and time slices, the northward expansion of WNV was projected. The expansion of WNV under the median scenario in the 2050s was 332,460 km^2^, representing a 1.6 fold increase from baseline conditions (no change under the cool, wet scenario in the 2020s, and a two fold increase under the warm, dry outcome scenario in the 2080s) (Table 4, Figure 3). WNV infection rate in the southern half of the Canadian prairies was generally higher than that in the north. Furthermore, most predicted areas of high WNV activity were located in the predicted grassland habitat for all selected outcome scenarios and time periods (Figure 3).

**Table 4:** Predicted distribution and range expansion of grassland habitat, the mosquito vector *Cx. tarsalis*, and WNV in the prairie provinces under current and future climate conditions. The predicted distribution of *Cx. tarsalis* and WNV was assumed to be limited by the availability of grassland habitat.

Outcome scenarios and time slices		Distribution area ^2^				Area expansion ^3^				Fold change ^4^		
		Grassland	*Cx. tarsalis*	WNV		Grassland	*Cx. tarsalis*	WNV		Grassland	*Cx. tarsalis*	WNV
**Current ^1^**		607,018	566,506	539,877								
**2010–2039**												
**Cool, wet**		543,502	536,042	516,619		−23,004	−30,464	−23,258		0.90	0.95	0.96
**Median**		727,509	727,029	711,578		120,491	160,523	171,701		1.20	1.28	1.32
**Warm, dry**		624,783	617,767	586,466		17,765	51,261	46,589		1.03	1.09	1.09
**2040–2069**												
**Cool, wet**		599,256	599,256	582,998		−7,762	32,750	43,121		0.99	1.06	1.08
**Median**		905,701	905,701	872,337		298,683	339,195	332,460		1.49	1.60	1.62
**Warm, dry**		1,198,242	1,198,242	1,151,876		591,224	631,736	611,999		1.97	2.12	2.13
**2070–2099**												
**Cool, wet**		664,150	664,150	657,321		57,132	97,644	117,444		1.09	1.17	1.22
**Median**		1,082,641	1,082,641	1,036,084		475,623	516,135	496,207		1.78	1.91	1.92
**Warm, dry**		1,449,128	1,449,128	1,263,070		842110	882,622	723,193		2.39	2.56	2.34

^1 ^The current distribution area of *Cx. tarsalis* and WNV are in the Canadian prairie ecozone based on the 1961–1990 climate condition. ^2 ^To estimate the distribution area of outcome scenarios, the availability of grassland habitat is set as a criterion for *Cx. tarsalis* in the prairie provinces. ^3 ^Area expansion = future distribution area (based on outcome scenarios) minus current distribution area. ^4 ^Fold change = future distribution area (based on outcome scenarios) divided by the current distribution area.

**Table 5:** Temporal distribution, mean abundance, log(y+1) transformed, and fold change of *Cx. tarsalis* abundance in the Canadian prairie ecozone for current and three future periods of selected outcome scenarios.

Outcome scenarios	May	June		July		August		September
	Abun (SD)	Abun (SD)	Fold change ^1^	Abun (SD)	Fold change	Abun (SD)	Fold change	Abun (SD)
Current	None	1.22 (0.33)		2.26 (0.32)		2.20 (0.32)		None
**2010–2039**								
Cool, wet	None	1.48 (0.32)	1.21	2.56 (0.29)	1.13	2.43 (0.31)	1.10	None
Median	None	1.57 (0.31)	1.29	2.68 (0.32)	1.19	2.75 (0.30)	1.25	0.28 (0.60)
Warm, dry	None	1.71 (0.31)	1.40	2.62 (0.28)	1.16	2.65 (0.30)	1.20	0.08 (0.33)
**2040–2069**								
Cool, wet	None	1.57 (0.29)	1.29	2.66 (0.26)	1.18	2.54 (0.31)	1.15	0.05 (0.28)
Median	0.05 (0.16)	1.97 (0.26)	1.61	3.03 (0.29)	1.34	3.02 (0.27)	1.37	1.11 (0.76)
Warm, dry	0.37 (0.39)	2.30 (0.32)	1.89	3.34 (0.34)	1.48	3.49 (0.32)	1.59	1.90 (0.50)
**2070–2099**								
Cool, wet	0.02 (0.09)	1.72 (0.33)	1.41	2.75 (0.28)	1.22	2.69 (0.30)	1.22	0.28 (0.63)
Median	0.49 (0.36)	2.26 (0.27)	1.85	3.32 (0.32)	1.47	3.39 (0.33)	1.54	1.83 (0.36)
Warm, dry	1.18 (0.27)	3.04 (0.31)	2.49	4.27 (0.32)	1.89	4.33 (0.31)	1.97	2.87 (0.26)

Abun: abundance of *Cx. tarsalis*; SD: standard deviation; None: no occurrence of *Cx. tarsalis*. ^1 ^Fold change = future abundance (based on outcome scenarios) divided by the current abundance of *Cx. tarsalis*.

**Table 6:** Temporal distribution and fold change of WNV infection rate (number of infected mosquitos per 1,000 mosquitoes) in *Cx. tarsalis* mosquitoes in the Canadian prairie ecozone for current and three future periods of selected outcome scenarios.

Outcome scenarios	May	June		July		August		September
	IR(SD)	IR (SD)	Fold change ^1^	IR (SD)	Fold change	IR (SD)	Fold change	IR (SD)
Current	None	0.54 (0.25)		1.33 (0.45)		2.23 (0.94)		None
**2010–2039**								
Cool, wet	None	0.51 (0.16)	0.94	1.14 (0.42)	0.86	2.88 (1.27)	1.29	None
Median	None	0.86 (0.25)	1.59	2.02 (0.96)	1.52	5.17 (2.64)	2.32	0.44 (1.32)
Warm, dry	None	0.67 (0.22)	1.24	1.67 (0.71)	1.26	2.84 (1.42)	1.27	0.40 (0.99)
**2040–2069**								
Cool, wet	None	0.72 (0.23)	1.33	1.62 (0.66)	1.22	3.20 (1.47)	1.43	0.06 (0.41)
Median	0.02 (0.09)	1.91 (0.71)	3.54	4.87 (1.91)	3.66	17.91 (10.0)	8.03	4.32 (4.63)
Warm, dry	0.17 (0.21)	1.37 (0.46)	2.54	5.91 (3.08)	4.44	18.08 (9.98)	8.11	10.18 (6.93)
**2070–2099**								
Cool, wet	0.004 (0.03)	1.01 (0.31)	1.87	1.91 (0.87)	1.44	3.74 (1.76)	1.68	0.28 (0.87)
Median	0.001 (0.001)	1.79 (0.64)	3.31	5.53 (2.57)	4.16	19.95 (11.81)	8.95	9.44 (7.06)
Warm, dry	0.61 (0.22)	2.70 (0.93)	5.00	17.55 (6.70)	13.20	61.21 (27.78)	27.45	30.89 (11.66)

IR: WNV infection rate in *Cx. tarsalis*; SD: standard deviation; None: absence of WNV in the *Cx. tarsalis*. ^1 ^Fold change = future WNV infection rate (based on outcome scenarios) divided by the current infection rate in *Cx. tarsalis*.

## 4. Discussion

Climate change is expected to influence the distribution of both vectors and vector borne pathogens, and contribute to the expansion or shifting of endemic regions [25,40,41]. This study demonstrates the potential for substantial expansion of the transmission season and geographic distribution of a recently introduced vector-borne disease in a highly endemic region of North America as a result of rapid climate and landscape change. 

We constructed models for predicting abundance of the primary mosquito vector *Cx. tarsalis* and WNV infection rate, and populated these with data from baseline and selected future climate scenarios to assess the effects of climate and landscape change on WNV in the Canadian prairie ecozone. Under even the most optimistic of scenarios, WNV will undergo northern range expansion and extension of the transmission season by the 2050s. Based on a middle-of-the-road scenario, approximately half to two/thirds of the northern portion of the prairie provinces will have a climate newly suitable for WNV transmission by the 2050s. Under the most extreme warming conditions, peak mosquito infection rates could be 30 times that of baseline, representing a substantial increase in infection pressure for people and animals alike.

Although higher temperatures may lead to increased mosquito mortality and thus represent a natural check on viral amplification, our results suggest that mean monthly temperatures will not exceed the upper threshold for survival of adult female *Cx. tarsalis*. In addition, mosquitoes may select cooler microhabitats if temperatures exceed tolerances. Therefore, the observed temporal and spatial distribution of WNV in the Canadian prairies will remain primarily determined by the lower temperature limitation for WNV amplification in *Cx. tarsalis* (estimated to be 14.3 °C) [37]. Laboratory experiments demonstrate that the temperature threshold for survival of *Cx. tarsalis* is generally between 14 °C and 35 °C, and within this range, temperature is positively correlated with development rate of vector [36,42]. Therefore, climate change will lead to higher development rates for vector without a compensatory increase in mosquito mortality. Moreover, increased temperatures will also increase the infection rate of WNV in *Cx. tarsalis*, especially in the southern part of the Canadian prairies.

Many factors besides climate are important determinants of the distribution and incidence of vector borne diseases, such as habitat suitability for competent vectors [43]. Changes in future climate could also induce shifts in habitat distribution and affect habitat suitability for vectors [25,40]. In the current study, we used the constructed model to predict the distribution of grassland habitat under current and selected future outcome scenarios. Northward expansion of grassland has been predicted in Western Canada in a future of climate change, with boreal forest replaced by aspen parkland and grassland, and current Canadian grassland types replaced by those found in the U.S. Great Plains [26,27,28]. These latitudinal shifts in vegetation zones will create more suitable habitat for *Cx. tarsalis* in the northern part of the prairie provinces, while maintaining suitable habitat in the current Canadian prairie ecozone. However, the spatial expansion of *Cx. tarsalis* and WNV distribution in the prairie provinces will lag behind the shifts of vegetation zones. 

The predicted distribution of grassland revealed another smaller area located in northern Alberta where the climate is appropriate for grassland habitat (Figure 1, Figure 2). Isolated grasslands resembling mixed prairie communities of the northern Great Plains are observed in this area [44,45,46]. In addition, recent studies have also revealed *Cx. tarsalis* in the region of these grassland remnants, which extend into the southern Northwest Territories, although no WNV was detected [11]. These empirical observations validated our prediction that if grassland habitat is available, *Cx. tarsalis* can already establish in the northern regions of the prairie provinces under current and projected future climate conditions; however, the activity of WNV remains low or nonexistent in these regions under current climate conditions. Moreover, the Canadian prairie represents the northernmost edge of WNV distribution in the western hemisphere. As *Cx. tarsalis*, WNV, and other arboviruses expand northward out of their current endemic area into regions where humans, domestic livestock, and wildlife lack immunity, these vector-borne diseases may emerge in these newly vulnerable populations [47,48].

Temperature increases and the ecological impact of climate change are predicted to be greater in temperate and polar regions than in tropical regions [47,49,50]. Increasing environmental temperature shortens the maturation time required for *Cx. tarsalis* and the extrinsic incubation period of West Nile virus. Furthermore, it also accelerates the mosquito gonadotrophic cycle and affects mosquito survival. Although beyond the scope of the current study, these relationships will influence virus transmission by increasing the contact rate between *Cx. tarsalis* and competent vertebrate hosts [37,51]. 

Although we have demonstrated that changing climate and habitat will drastically alter the current distribution and abundance of a newly-introduced vector-borne disease, a number of other factors will also affect the ecology of WNV, and will in turn be affected by climate conditions. These factors include ability of hosts to migrate, disperse and adapt to changing local environments, host resistance to disease, biotic interactions, evolutionary change, other anthropogenic alternations of environment, and efforts of disease control [25,52]. Future models addressing how these factors will affect the ecology of WNV are critically needed. In addition, climate change predictions are themselves subject to uncertainty in terms of the magnitude and scale of physical and socioeconomic drivers, which will need to be addressed to more accurately predict changes in the ecology of vectors and vector-borne diseases. Finally, the precise lag time of habitat change (*i.e.*, from boreal forest to grassland) and subsequent dispersal of vectors and hosts to newly suitable habitat remain unclear. Therefore, in order to validate our predictions and improve the predictive ability of these models, further monitoring of distribution and abundance of *Cx. tarsalis* and WNV is recommended, especially in regions that we have identified as vulnerable to range expansion and enhanced endemic amplification within the next 20–100 years.

## 5. Conclusions

The present study evaluated the potential effects of future climate and landscape change on the increased distribution and abundance of a newly-introduced vector-borne disease (WNV) and its primary vector (*Cx. tarsalis*) in a highly endemic region of North America. Studies like this one that use predictive models based on recorded data, known biological thresholds, and the best available climate scenarios covering the full range of outcomes provide vital information for public health professionals and policy makers to set priorities for mitigation and adaptation in the near future.
